# The Pan-Cytokeratin Staining Intensity and Fractal Computational Analysis of Breast Tumor Malignant Growth Patterns Prognosticate the Occurrence of Distant Metastasis

**DOI:** 10.3389/fonc.2018.00348

**Published:** 2018-08-30

**Authors:** Nemanja Rajković, Xingyu Li, Konstantinos N. Plataniotis, Ksenija Kanjer, Marko Radulovic, Nebojša T. Milošević

**Affiliations:** ^1^Department of Biophysics, School of Medicine, University of Belgrade, Belgrade, Serbia; ^2^Multimedia Laboratory, The Edward S. Rogers Sr. Department of Electrical & Computer Engineering, University of Toronto, Toronto, ON, Canada; ^3^Department of Experimental Oncology, Institute for Oncology and Radiology, Belgrade, Serbia

**Keywords:** breast cancer, tumor, prognosis, metastasis, image analysis, fractal, pan-cytokeratin, histopathology

## Abstract

Improved prognosis of breast cancer outcome could prolong patient survival by reliable identification of patients at high risk of metastasis occurrence which could benefit from more aggressive treatments. Based on such clinical need, we prognostically evaluated the malignant cells in breast tumors, as the obvious potential source of unexploited prognostic information. The patient group was homogeneous, without any systemic treatments or lymph node spread, with smaller tumor size (pT1/2) and a long follow-up. Epithelial cells were labeled with AE1/AE3 pan-cytokeratin antibody cocktail and comprehensively analyzed. Monofractal and multifractal analyses were applied for quantification of distribution, shape, complexity and texture of malignant cell clusters, while *mean pixel intensity* and *total area* were measures of the pan-cytokeratin immunostaining intensity. The results surprisingly indicate that simple binary images and monofractal analysis provided better prognostic information then grayscale images and multifractal analysis. The key findings were that shapes and distribution of malignant cell clusters (by *binary fractal dimension*; AUC = 0.29), their contour shapes (by outline fractal dimension; AUC = 0.31) and intensity of the pan-cytokeratin immunostaining (by *mean pixel intensity*; AUC = 0.30) offered significant performance in metastasis risk prognostication. The results reveal an association between the lower pan-cytokeratin staining intensity and the high metastasis risk. Another interesting result was that multivariate analysis could confirm the prognostic independence only for fractal but not for immunostaining intensity features. The obtained results reveal several novel and unexpected findings highlighting the independent prognostic efficacy of malignant cell cluster distribution and contour shapes in breast tumors.

## Introduction

The primary breast tumor is not life-threatening until the disease turns into a systemic condition by distant tissue invasion. Such distant recurrence or metastasis has an overall rate of up to 50% during 20 years after surgery (1). Besides local surgery and radiation, patients are therefore also treated with systemic cytotoxic therapy in an attempt to eliminate any distant micrometastatic malignant spread. Taken together, the goal to achieve personalized therapeutic protocols based on reliably prognosticated individual risk is the main clinical applicability of metastasis risk prognosis improvement. The benefit to the patient is to maximize a therapeutic response and minimize toxicity.

The current breast cancer prognosis is based on the clinicopathological evaluation (TNM classification, histological features, age, menopause), receptor status (hormone receptors and HER2) (2) and molecular signatures such as Mammaprint and OncotypeDX (3). Unfortunately, the mentioned current prognostic classifications including the costly molecular signature tools are still not sufficiently reliable with 65%, accuracy and AUC of 0.69 (4). For this reason, computational tumor histomorphology analysis is developing as another approach aimed to achieve prognostic improvement in breast cancer (5–8). This methodology exploits information from histopathology images that cannot be quantified by the conventional microscopic examination, such as distribution, texture and shape. Furthermore, the main advantages of this approach include its high speed, cost-efficiency and convenience based on the widespread availability of histopathology specimens in digital form. Algorithms exploited for analysis of medical images comprise statistical (co-occurrence), geometrical/structural (Voronoi tessellation, fractal), model-based (Markov random fields) and spectral/signal processing (Gabor filters, wavelet transform and curvelets).

The histomorphological diversity of tumors has been exploited for prognostic purposes since the mid-nineteenth century (9). It was later confirmed that primary tumor is indeed the site of metastatic cell dissemination which further supported its importance as the source of prognostic information (10). Therefore, this study focuses primarily on the analysis of malignant cell growth patterns of primary breast tumors. Such structures can be observed on pan-tissue hematoxylin and eosin (H&E) stained tumor sections but are easier to investigate upon pan-cytokeratin immunostaining because breast tumors mostly arise by neoplastic transformation of epithelial cells. The pan-cytokeratin antibody has been mainly exploited in prognostic studies of circulating and disseminated epithelial tumor cells, particularly in assessing sentinel lymph node biopsies in breast cancer (11). Other approaches comprised prognostic investigations of tumor immunostaining by cytokeratin panels (12) or single keratins (13).

Epithelial cells within breast tumors immunostained for pan-cytokeratins have been previously prognostically evaluated using GLCM and binary monofractal algorithms (14–16). This study extends the previous reports by undertaking a much wider investigation by utilizing the monofractal, multifractal and intensity analysis of binary and grayscale images of pan-cytokeratin stained malignant cell clusters. Fractal geometry is well-suited for quantification of the distribution, shape, texture and complexity of chaotic malignant cell growth (17).

Taken together, we set out to perform the first intensity evaluation of malignant epithelial cell immunostaining in combination with their exhaustive distribution, shape, texture and complexity assessment.

## Materials and methods

Writing of this report was done to include all relevant experimental detail according to recommendations for tumor marker prognostic studies (18).

### Ethics approval statement

The study was approved by the Institutional Review Board (Belgrade University, School of Medicine, approval #29/VI-4) and conforms with The Code of Ethics of the World Medical Association (Declaration of Helsinki), printed in the British Medical Journal (July 18, 1964) and its 7th revision in 2013.

### Patient group

Selection of breast cancer patients was retrospective, based on the absence of hormonal, cytotoxic or other systemic treatments (natural course of the disease) according to recommendations valid in the year 1993 for the primary operable breast carcinoma with pT1/2, grade 1/2, without lymph node involvement or metastasis (N0M0). All patients were treated locally by surgery and radiation. Patient data were received in a de-identified form without direct or indirect identifiers that could enable re-identification (Safe-Harbour methodology of the 2012 Health Insurance Portability and Accountability Act). All patients were female Caucasian from the Republic of Serbia, treated in the same year (1993) and in the single institution (Institute of Oncology and Radiology of Serbia). Based on the 10 fmol/mg and 20 fmol/mg respective cutpoints, 76% of patients were positive for estrogen receptor (ER, median of 32 fmol/ml) and 24% were positive for the progesterone receptor with a median of 6 fmol/ml. The dextran-coated charcoal method was used for estrogen and progesterone receptor assay as previously described in detail (19). HER2 status was available for 52 of the 73 patients used in this study and was positive in 21% of the patients. The prospective sample size was calculated by taking into account the event rate, a median standard deviation between high- and low-risk groups and effect size (hazard ratio). These parameters were estimated by a pilot study on 35 samples, prior to the full experiment. For a target power of 0.8, the distance between means of feature values for the high- and low-risk groups of 0.52 standard deviations, minimal effect size of 0.14 > HR > 7.0, alpha 0.05 and an event rate of 25%, the required numbers were 31 patients with 10 events (Stata/MP 13 package, StataCorp, College Station, TX). The final sample size was 73 patients with 17 events. The sample size reflects a rarity of the systemically untreated breast cancer patients and the strict avoidance of missing data. The actual event rate was 23%, the median standard deviation of texture variables was 0.54 and the average effect size 25.4. The *post-hoc* power analysis revealed the actual power of 1.0. The median age at diagnosis was 57 years (range 41–80). The median follow-up time to metastasis was 38 months, ranging between 16 and 145 months, while the median follow-up time for patients without metastasis was 147 months by a reverse Kaplan-Meier method, ranging between 77 and 165 months. The Adjuvant! Online score (Adjuvant group Inc., NJ, USA) for Breast Cancer (Version 8.0) was calculated at the Adjuvant! Online site as a 10-years risk of relapse with no additional therapy based on age, tumor grade, estrogen receptor status and tumor size (20).

### Image analysis workflow

The methodological process included immunostaining, selection of tissue sections, image acquisition, stain decomposition, image analysis, data categorization and prognostic evaluation with validation. These steps are respectively described under the subheadings below.

### Immunostaining

Tissue of invasive primary breast tumors was obtained during surgery. The tissue was formalin-fixed, paraffin-embedded and cut to produce 4 μm whole sections which were bonded on clean glass slides. Freshly cut whole tissue sections were immunostained without counterstain in order to highlight only epithelial cells. A heat-mediated antigen retrieval step was performed in EDTA pH 8 buffer with a water bath set at 95°C for 40 min. Endogenous peroxidase was quenched with 3% H_2_O_2_ in methanol for 30 min. Five percent goat serum was used for the 1 h pre-incubation step. The whole tissue sections were incubated with the CD8 rabbit monoclonal primary antibody (ThermoFisher Scientific, Waltham, MA; #RM-9116-S1), followed by the monoclonal mouse anti-human pan-cytokeratin primary antibody clones mAE1/AE3 (Dako, Glostrup, Denmark, #M3515) in 5% goat serum for 60 min. Sections were washed in PBS and incubated with secondary goat anti-rabbit IgG HRP conjugate (Jackson ImmunoResearch Laboratories, West Grove, PA; # 111-035-144) followed by polyclonal goat anti-mouse IgG alkaline phosphatase conjugate (Southern Biotech, Birmingham, AL; #1030-04) in 5% goat serum. Following washes in PBS, nickel-enhanced DAB (Vector Laboratories, Burlingame, CA) and subsequently the Fast Blue RR (Sigma-Aldrich, St. Louis, MO) were used as chromogens. AE1/AE3 antibody cocktail mainly stains epithelial cells by detecting cytokeratins 1–8, 10, 14–16 and 19.

### Selection of tissue sections

For maximal reproducibility and validity, the pathologist (KK) has selected the sections containing the most characteristic growth patterns for each individual tumor, with the highest content of pan-cytokeratin stained malignant cells and without any artifacts or normal structures. Normal and malignant cell arrangements stained with pan-cytokeratin were identified morphologically.

### Image acquisition

Color images of the immunostained tumor histopathology slides were acquired by use of the NanoZoomer Hamamatsu-XRC12000 high-resolution digital slide scanner (Hamamatsu City, Japan).

### Stain decomposition

Due to the fact that tissue sections were double stained for pan-cytokeratin (blue) and CD8 (brown), it was necessary to decompose images into single-stained channels, each containing only one chemical dye as previously described in detail (21). Specifically, the stain decomposition algorithm separates the immunostaining images into CD8 and pan-cytokeratin channels. All downstream image analysis in this study was performed in the resulting pan-cytokeratin channels.

### Image analysis

Color images were transformed to grayscale format by the *run(“8-bit”)* command of the *Fiji*/*ImageJ* version 1.52b, an open platform for biomedical image analysis (22). Images were further converted to a binary format by the *run(“Make Binary”)* command of Fiji/ImageJ. The obtained grayscale and binary image formats were analyzed for staining intensity. Furthermore, monofractal and multifractal algorithms were applied for extraction of numerical measures (features) for each image based on analysis of the pixel information.

The staining intensity features are simple to calculate. The *mean pixel intensity* is an average value of grayscale pixel intensities, ranging from 0 to 255 for 8-bit images, calculated by the “*measure*” function of ImageJ. The *total area* of immunostaining was calculated by use of automatically thresholded binary images and the “*analyze particles*” function of ImageJ.

Calculation and interpretation of fractal features is more complicated. Fractal dimension is the main monofractal feature. It is primarily a measure of complexity but depending on the type of analysis it can also be sensitive to object shapes, texture and distribution. In the fractal analysis, complexity refers to a change in detail (foreground pixels) with a change in scale (magnification). Multifractal theory can be considered an extension of the monofractal approach, particularly if an image contains an uneven distribution of complexity with fractal dimension varying across an image (23). The multifractal analysis delivers spectrums of fractal dimensions such as *D*_*Q*_ vs. α and *f(*α*)* vs. *Q*, thus providing more information about an image in comparison to a single monofractal dimension.

Monofractal and multifractal analysis of binary images was performed by use of the regular non-overlapping box counting method (FracLac plugin version *2015Sep090313a9330* for *Fiji*/*ImageJ*) according to formulas previously described in full detail (24). The box-counting method involves overlaying of the binary image with a meshed lattice of square boxes of decreasing size (scale) ε expressed as the box size relative to image size. The space filling properties of the image by box counting is calculated by considering the relationship between the numbers of boxes containing at least one forward pixel (non-empty boxes) and box sizes.

Monofractal and multifractal analyses of grayscale images were performed by use of the box counting method adjusted for grayscale images (differential box counting, FracLac plugin for ImageJ). While regular box counting for binary images only considers presence or absence of foreground pixels within the box, differential box counting for grayscale images calculates the difference in intensity of the pixels in a box at each box size (ε) as explained previously in full detail (24).

Monofractal features calculated for binary and grayscale images included *FD, FD*_outline_ and Λ. Multifractal features provided by FracLac software were: α, *f(*α*)*, and *D*_*Q*_, for the range of 200 *Q* values from −10 to +10 in 0.1 increments. By use of Excel formulas, ten additional multifractal features were calculated: *D*_*Qmax*_, *f(*α*)*_*min*_, *f(*α*)*_*max*_, α *corresponding to f(*α*)*_*min*_, α *corresponding to f(*α*)*_*max*_, *slope D*_*Q*_*(Q), slope* α*(Q), slope f(*α*)(Q), slope DQ(Q)(*–*1 to 3), f(*α*) summed for Q* > *0*. Taken together, 3 monofractal and 13 multifractal features were calculated, each for grayscale and binary images, a total of 32 fractal features.

### Data categorization and prognostic evaluation

The ROC and Cox regression analyses were used as prognostic evaluation tools for the clinicopathological, intensity and fractal features. Discrimination is the capability of prognostic classifiers to stratify patients with and without metastasis. The area under the rate of change curve (AUC) was employed as a quantitative measure of discrimination efficiency, calculated by use of continuous feature values. AUC chance accuracy is defined at 0.5, while perfect accuracy equals 0.0 or 1.0. The discrimination efficiency increases farther from 0.5, whereby 0.4 or 0.6 is considered as the fair discrimination performance, 0.3 or 0.7 as good, 0.2 or 0.8 as excellent, and 0.1 or 0.9 as almost perfect.

Data categorization by use of a cutpoint divided patients into low- and high-risk subgroups and was necessary for the Cox proportional hazards regression test. The optimal cutpoint selection was performed by *X-tile 3.6.1* software (Yale University, New Haven, CT). As the data categorization step may introduce bias, ROC analysis and Cox regression often disagree in their prognostic evaluation. Cox proportional hazards regression test compares the predicted and actual metastasis outcomes. The proportional hazards assumption was satisfied for each feature based on the Schoenfeld residuals by phtest (Stata/MP 13 package, StataCorp, College Station, TX). The hazard ratio (HR) is the effect size of the Cox regression reflecting the metastasis rates in high- and low-risk groups of patients. It indicates chance performance at HR = 1.0. Multivariate Cox proportional hazards regression analysis tested the independence of each prognostic factor. Variables categorized by outcome were added to a full model using forward selection entry criterion of *P* < 0.20 in univariate analysis and removed using backward elimination according to a selection stay criterion of *P* < 0.05. The IBM SPSS Statistics 23 (IBM Corp. Armonk, NY) was employed for bootstrap-corrected Cox analysis. Spearman's correlation coefficients were calculated by Statistica 12 (Tibco Software Inc. Paolo Alto, CA).

Kaplan–Meier survival analysis was completed for the period from diagnosis to metastasis manifestation (IBM SPSS). Spearman's rank correlation test was used for evaluation of the association strengths between pairs of variables (Statistica 12).

### Validation

The over-optimism of the ROC (Stata/MP 13) and Cox (IBM SPSS) analysis was corrected by the bootstrap internal validation with 1,000 data resamples (25).

## Results

This study provides prognostic comparison of the simple immunostaining intensity with the more perplexing fractal analysis assessing staining distribution, shapes, complexity and texture. Grayscale and binary image formats were used for analysis of the pan-cytokeratin immunostained breast tumor malignant cell clusters.

**Figure 1:**
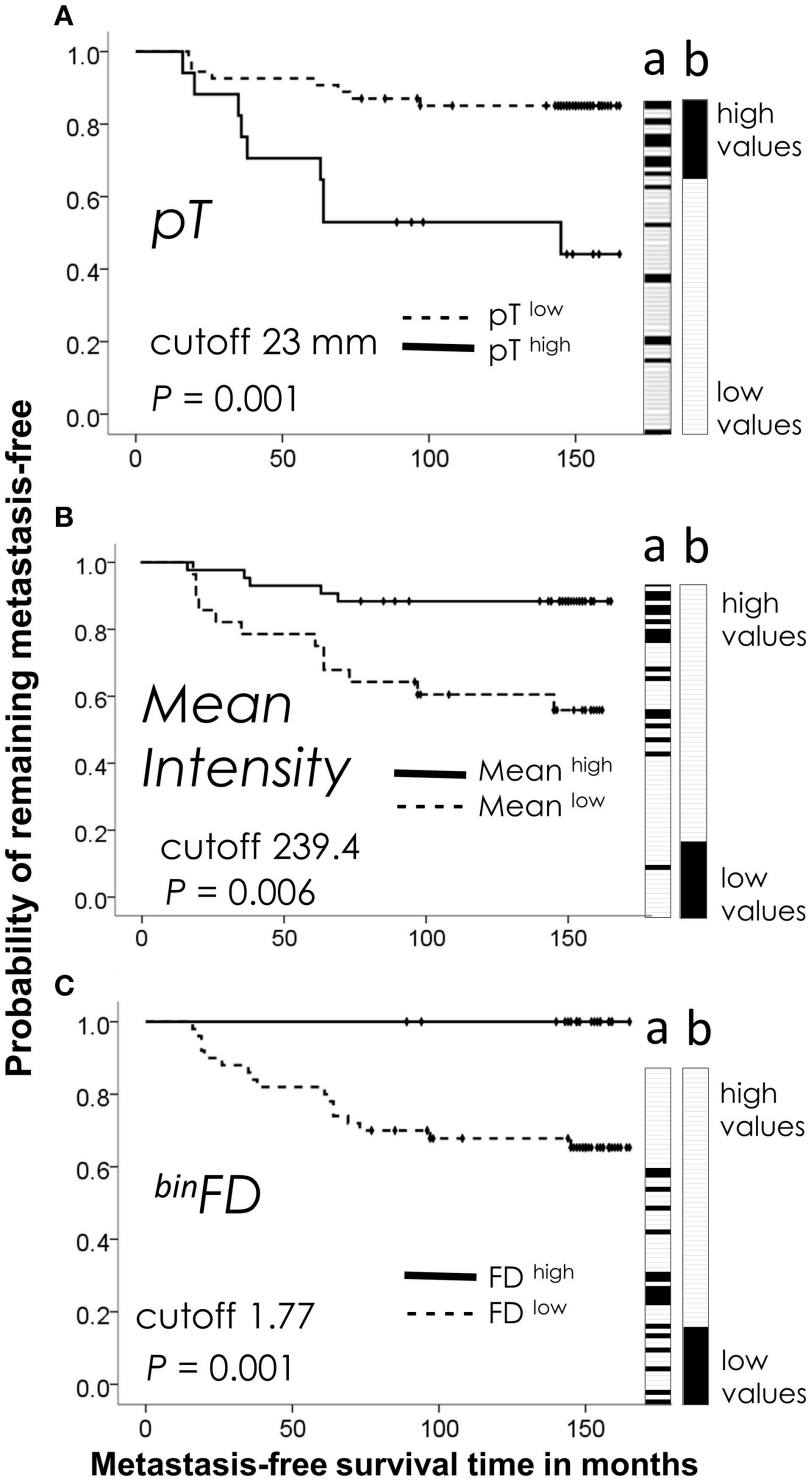
Kaplan-Meier analysis of the best performing feature from each group: **(A)** clinicopathological (tumor size, *pT*), **(B)** immunostaining intensity (*mean* pixel intensity), **(C)** fractal (^*bin*^*FD*). Plots reveal prognostic discrimination efficiencies of feature values categorized by indicated cutpoint values. Dotted lines show the patient subgroup with lower feature values (below the cutpoint, feature^low^). Feature^high^ value subgroup is plotted on solid lines. (a) Ordering of patients by the ascending continuous values of each feature. Patients with metastasis are indicated by black tiles and patients without metastasis by white tiles. Lane a thus illustrates the prognostic performance of each feature to stratify the patients (a) into high and low risk groups by their continuous values, while Kaplan-Meier plots indicate the prognostic performance after categorization of feature values. (b) The ideal stratification of the actual metastasis occurrence is shown for comparison. The time refers to the interval from a primary breast tumor surgery until the occurrence of first distant metastasis or end of follow-up. *P*-values were calculated by the Cox proportional hazards regression.

**Figure 2:**
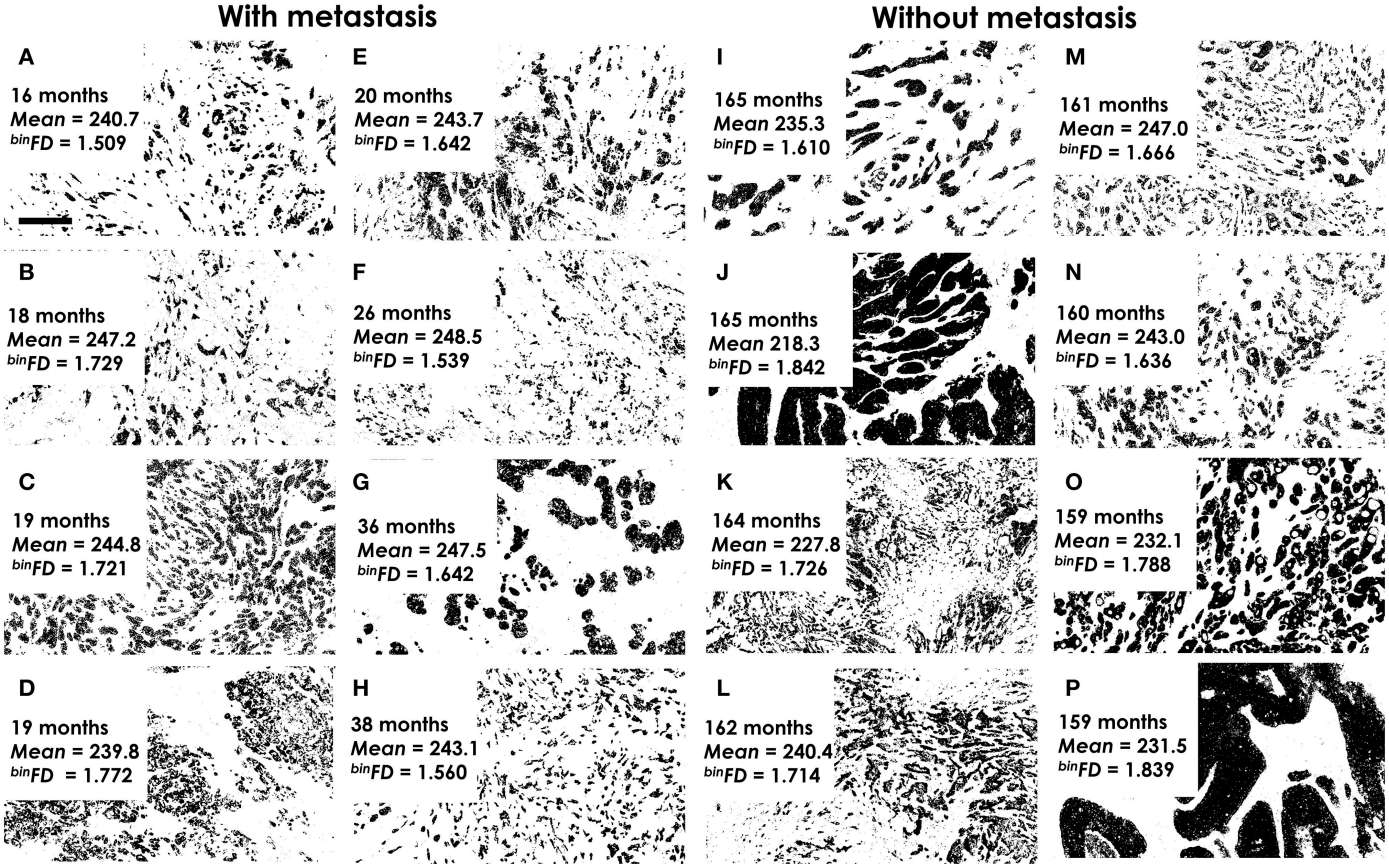
Examples of analyzed binary histological images. **(A–H)** The highest-risk patients with quickest metastasis appearance. **(I–P)** The lowest-risk patients, without metastasis and the longest follow-up. The time to metastasis **(A–H)** or end of follow-up **(I–P)**, mean pixel intensity (*mean*) and *fractal dimension* values (^*bin*^*FD*) are indicated for each image. It is important to note that lower pixel intensity values actually indicate darker graylevels. Furthermore, the indicated mean pixel intensity values refer to graylevel images, not the presented binary images. All analyzed images represent areas of tumor tissue that are predominantly populated by malignant cells. Magnification ×400. Pixel size: 145 nm. Bar 50 μm, indicated on **(A)**.

Calculations of the staining-intensity, fractal and multifractal features are described in the Methods section. ROC analysis and univariate Cox proportional hazards regression test were the statistical tests employed for prognostic evaluation of the calculated features (Table 1). Furthermore, the prognostic performance was illustrated by Kaplan-Meier plots (Figure 1) and characterized by multivariate analysis (Table 2). Correlations of the calculated features are presented in Table 3. Examples of the analyzed binary images are presented in Figure 2.

**Table 1:** The prognostic significance of the clinicopathological, staining intensity, monofractal, and multifractal features.

**Parameter**	**Hazard ratio^a^**	**95% CI^a^**	***P-value*^a^**	**AUC^b^**	**95% CI^b^**	***P-value*^b^**
**CLINICOPATHOLOGICAL**						
Adjuvant!	4.6^*^	1.7–14.0	0.001	0.57	0.41–0.77	0.19
Tumor size	4.6^*^	1.73–13.5	0.002	0.64	0.44–0.83	0.14
ER	3.2^*^	1.3–9.0	0.009	0.61	0.44–0.78	0.13
**STAINING INTENSITY**^c^						
Mean int.	*O*.23^*^	0.05–0.61	0.006	0.30^*^	0.17–0.45	0.01
Total area	0.19	0.03–0.78	0.07	0.37	0.22–0.52	0.15
**MONOFRACTAL BINARY**						
*^*bin*^FD_*outline*_*	0.03^*^	0.02–0.04	0.001	0.31^*^	0.20–0.47	0.02
*^*bin*^FD*	0.03^*^	0.02–0.03	0.001	0.29^*^	0.21–0.46	0.01
*^*bin*^Λ*	8.8^*^	2.2–53.5	0.03	0.65^*^	0.51–0.78	0.05
**MULTIFRACTAL BINARY**						
*^*bin*^f(α)max*	24.1^*^	21.5–27.7	0.001	0.38	0.24–0.58	0.12
*^*bin*^α f(α)max*	0.04^*^	0.03–0.05	0.001	0.36	0.20–0.53	0.09
*^*bin*^slope fα (Q)*	0.02^*^	0.01–0.42	0.03	0.37	0.21–0.52	0.10
*^*bin*^f(α)sum Q > 0*	0.24^*^	0.02–0.80	0.03	0.35^*^	0.21–0.48	0.04

Bootstrap-corrected ROC analysis and Cox proportional hazards regression were used for evaluation of the prognostic significance.

* P ≤ 0.05.

a Cox proportional hazards regression analysis was performed by use of categorized data, bootstrap corrected.

b ROC analysis was performed by use of continuous data, with bootstrap correction.

c Mean intensity and total area were respectively calculated by use of grayscale and binarized images. *CI, confidence interval; AUC, area under the ROC curve; ER, estrogen receptor, Mean int., mean pixel intensity; ^bin^FD, binary fractal dimension; ^bin^Λ, binary lacunarity*.

**Table 2:** Multivariate Cox proportional hazards regression analysis.

	**Coefficient**	***P*-value^a^**	**HR**	**95% CI^a^**
Adjuvant! online	1.29	0.001	3.6	1.5–12.2
ER	0.85	0.04	2.3	0.89–6.9
*^*bin*^FD*	−12.43	0.001	0.00	0.00–0.00
*^*bin*^slope f(α)(Q)*	−2.28	0.02	0.10	0.00–0.47
*^*bin*^α fα(max)*	−11.4	0.006	0.00	0.00–1.9

Multivariate analysis was performed by inclusion of all significant predictors to capture their predictive redundancy.

a Bootstrap corrected.

*HR, hazard ratio; CI, confidence interval; ER, estrogen receptor; ^bin^FD, binary fractal dimension*.

**Table 3:** Correlations between the prognostically significant clinicopathological, intensity and fractal features.

**Features**	**Clinicopathological**			**Intensity**		**Monofractal**			**Multifractal**			
	**Adj**.	**pT**	**ER**	**Area**	**Mean**	***^*bin*^FD***	***^*bin*^FD_*out*_***	***^*bin*^FD***	***f(α)*_max_**	***α f(α)_*max*_***	***f(α) Q > 0***	***slope f(α)(Q)***
*Adjuvant!*	1.00	0.90^*^	0.20	0.07	−0.12	−0.14	−0.20	0.09	−0.05	0.02	−0.01	0.01
*pT*	0.90^*^	1.00	0.20	0.10	−0.08	−0.12	−0.15	0.08	−0.08	0.05	−0.01	−0.01
*ER*	0.20	0.20	1.00	−0.20	−0.01	−0.17	−0.10	0.06	0.07	−0.23	−0.20	−0.11
*Area*	0.07	0.10	−0.20	1.00	−0.69^*^	0.77^*^	0.60^*^	−0.80^*^	0.06	0.04	0.89^*^	0.89^*^
*Mean Int*.	0.12	0.08	0.01	0.69^*^	1.00	0.56^*^	0.43^*^	−0.65^*^	−0.05	−0.03	0.69^*^	0.70^*^
*^*bin*^FD*	−0.14	−0.12	−0.17	0.77^*^	−0.56^*^	1.00	0.93^*^	−0.81^*^	0.13	0.10	0.83^*^	0.82^*^
*^*bin*^FD_*outline*_*	−0.20	−0.15	−0.10	0.60^*^	−0.43^*^	0.93^*^	1.00	−0.71^*^	0.14	0.04	0.68^*^	0.68^*^
*^*bin*^FD*	0.09	0.08	0.06	−0.80^*^	0.65^*^	−0.81^*^	−0.71^*^	1.00	−0.16	0.23	−0.81^*^	−0.89^*^
*^*bin*^f(α)_*max*_*	−0.05	−0.08	0.07	0.06	0.05	0.13	0.14	−0.16	1.00	0.06	0.14	0.16
*^*bin*^α f(α)_*max*_*	0.02	0.05	−0.23	0.04	0.03	0.10	0.04	0.23	0.06	1.00	0.02	−0.12
*^*bin*^f(α) Q > 0*	−0.01	−0.01	−0.20	0.89^*^	−0.69^*^	0.83^*^	0.68^*^	−0.81^*^	0.14	0.02	1.00	0.95^*^
*^*bin*^Slope f(α)(Q)*	0.01	−0.01	−0.11	0.89^*^	−0.70^*^	0.82^*^	0.68^*^	−0.89^*^	0.16	−0.12	0.95^*^	1.00

Spearman's rank correlation coefficients are displayed.

* P ≤ 0.05.

*Adj., Adjuvant! online score; pT, tumor size; ER, estrogen receptor; Area, total stained area; Mean Int., mean immunostaining intensity; ^bin^FD, binary fractal dimension; ^bin^Λ, binary lacunarity*.

By the ROC analysis criteria, ^*bin*^*FD* provided the best overall prognostic performance with AUC of 0.29, followed by *Mean* pixel intensity and ^*bin*^*FD*_*outline*_ (Table 1). The other prognostically significant features by this criterion were *Adjuvant! Online score, tumor size* and *total area*. Only the features which showed prognostic significance by criteria of either ROC or Cox regression are presented in Table 1, while the 32 calculated fractal features are listed in the *Methods* section. Strikingly, none of the fractal features obtained on grayscale images could provide significant prognostic performance.

The prognostic performance evaluations by ROC and Cox regression statistical tests were often in disagreement. For instance, several features which were not prognostically significant by the criteria of ROC analysis emerged as significant by the Cox proportional hazards regression (Table 1). This may be ascribed to a stricter prognostic evaluation by ROC analysis. Furthermore, the outcome-based categorization of data for analysis by Cox regression often introduces the prognostic bias (26). It is also of note that Cox regression considers the time to the event while ROC analysis does not.

Among feature groups, the most distinct AUCs were achieved by the binary monofractal (AUC ranging between 0.29 and 0.31) and immunostaining intensity (0.35–0.70) groups (Table 1). The most pronounced prognostic HRs and the narrowest confidence intervals (95% CI) were noted for binary monofractal (HR ranging between 0.03 and 8.8) and binary multifractal groups (0.02–24.1, Table 1).

AUC and HR values do not only indicate the strength of association with metastasis outcome but also its direction. Thereby, AUC < 0.5 or HR < 1.0 indicate an association with low-risk, while AUC > 0.5 or HR > 1.0 indicate an association with high-risk of metastasis occurrence. ^*bin*^Λ and ^*bin*^*f(*α*)max* thus associated with high metastasis risk (Table 1). This simply means that patients with higher feature values are more likely to incur metastasis than patients with lower values. On the other hand, *mean pixel intensity*, ^*bin*^*FD*_*outline*_, ^*bin*^*FD*, ^*bin*^α *f(*α*)*_*max*_, ^*bin*^*slope f*α*(Q)*, ^*bin*^*f(*α*)* and ^*bin*^*sum Q* > *0*, all associated with low metastasis risk (Table 1).

Among the group of 55 estrogen receptor positive (ER+) patients, based on the standard 10 fmol/mg cutpoint, prognostic evaluation was very similar for each feature (not shown) to that indicated for the entire patient group in Table 1. This result indicated that prognostic performance of the examined staining intensity and fractal features was not ER-dependent. The prognostic evaluation could not be performed in the ER-negative subgroup due to its small size of 18 patients, below the calculated minimal sample size of 31 patients.

The multivariate analysis presented in Table 2 was performed by use of all features that showed prognostic significance. ^*bin*^*FD* thereby emerged as the prognostically most important variable, based on its coefficient, *P*-value, *HR* and the narrowest *CI 95%* (Table 2).

Several prognostically significant individual features were highly correlated (Table 3). The highest correlations were surprisingly observed between the *total immunostained area* as the simple feature depending on the number of black pixels and the elaborate fractal parameters such as ^*bin*^*FD*, ^*bin*^*f(*α*) Q* > *0* and ^*bin*^Λ (with Spearman's coefficients of up to 0.89; Table 3). This result indicated that several fractal features to a great extent reflect the simple count of black pixels within an image. Among feature groups, clinicopathological parameters showed almost no correlation with the other groups while immunostaining intensity, monofractal and multifractal feature groups exhibited a number of highly pronounced mutual correlations (Table 3). Spearman's coefficients for feature correlations between immunostaining intensity and monofractal feature groups ranged from 0.43 to 0.80 and from 0.24 to 0.89 for immunostaining intensity and multifractal groups (Table 3). High correlations were also observed within the feature groups, for instance: tumor size and *Adjuvant! Online score* (0.90), ^*bin*^*FD* and ^*bin*^*FD*_outline_ (0.93), ^*bin*^*FD* and ^*bin*^Λ (−0.81), ^*bin*^*slope f(*α*)(Q) and* ^*bin*^*f(*α*) Q* > *0* (0.95, Table 3). Overall, the *total stained area* was the feature mostly correlated with other features as its total sum of significant absolute Spearman's coefficient values amounted to 5.5, followed by ^*bin*^*FD* with a sum of 5.37, ^*bin*^Λ (5.24) and *mean pixel intensity* (4.26).

Kaplan-Meier plots illustrate discrimination efficiencies of the best-performing features from each group: *pT* for the clinicopathological group (Figure 1A), *Mean* pixel intensity for the immunostaining intensity group (Figure 1B) and ^*bin*^*FD* for the fractal group (Figure 1C). These plots illustrate the prognostic performance by the categorized feature values. Categorization was performed by use of a cutpoint to divide the measured continuous feature values into high- and low-risk patient subgroups. Figure 1 also illustrates the prognostic performance of continuous values prior to their categorization (Figure 1, lane a). The black tiles indicate patients with metastasis and white tiles without metastasis. When sorted in ascending order, these values tend to order the patients according to their recorded actual metastasis occurrence, with black tiles pre-dominating at one side and white tiles pre-dominating at the opposite side (Figure 1, lane a). The ideal separation of the actual metastasis risk is shown for comparison in Figure 1, lane b. Examination of the Figure 1 indicates that high values of ^*bin*^*FD* define a homogeneous low-risk group of 21 patients, with a low-risk upper curve entirely flat (Figure 1C) and the all-white tile region in the high-value range (Figure 1C, lane a). This indicates a better stratification of low-risk patients for ^*bin*^*FD* and to an extent also for the mean pixel intensity feature (Figure 1, lane a). High-risk patients are clearly stratified at the opposite end, but this stratification does not approach homogeneity.

Examples of the pan-cytokeratin stained tumor sections for patients at the actual metastasis risk extremes are presented in Figure 2. The eight patients with soonest (16–38 months) metastasis occurrence were considered at highest risk (Figures 2A–H). The lowest risk patients included those which remained metastasis-free even during the longest follow-up periods (Figures 2I–P). The fractal dimension (or complexity) of a line is 1 and of a filled square 2. This means that fractal dimension values for a two-dimensional image must range between 1 and 2. The actual range of the ^*bin*^*FDs* within the studied group of 73 patients was 1.54–1.87 (Figure 2). The mean pixel intensity values ranged (Figure 2) between 219.5 (darkest) and 248.3 (lightest). The respective average values for the high- and low-risk groups were 244.5 ± 3.1, 234.4 ± 9.1 for *mean* intensity and 1.640 ± 0.1, 1.722 ± 0.09 for ^*bin*^*FD*. It is important to note that lower pixel intensity values actually indicate darker graylevels. The presented images thus illustrate that darker immunostaining and higher ^*bin*^*FD* values indicate lower metastasis risk.

## Discussion

We report the first prognostic evaluation of immunostaining by exhaustive assessment of its intensity, distribution, shapes and texture. This investigation was aimed at improvement of breast cancer disease course prognostication.

Whereas the prognostic value of malignant cell distribution in breast tumors has been previously established (14–16), this is the first investigation of the intensity of their immunostaining for pan-cytokeratin. We found that the *mean intensity* of immunostaining significantly associated with low-risk. Interestingly, based on this result it can be concluded that tumors at high-risk of metastatic dissemination typically presented lower intensity of pan-cytokeratin staining in comparison to low-risk tumors. Such a decrease might be explained by downregulation of cytokeratins. This study was designed to compensate for the possible variation in the expression of individual cytokeratins by choosing the AE1/AE3 antibody preparation with a wide range of binding to 13 cytokeratins. The observed lower pan-cytokeratin staining intensity in high-risk tumors was in agreement with the previous report of complete loss of such staining for malignant cells disseminated to lymph nodes, probably associated with cell dedifferentiation (27). The advantage of the current study was its assessment of the pan-cytokeratin staining intensity, however, malignant cells not stained by pan-cytokeratin could not be quantified. The malignant cells could have been morphologically identified on hematoxylin-eosin counterstained sections even if unstained by the pan-cytokeratin antibody. However, histology sections used in this study were not counterstained in order to avoid any interference with the analyzed pan-cytokeratin immunostaining.

Another immunostaining intensity parameter measured in this study was the *total area* of immunostaining which reflected the number of epithelial cells. It is important to note that none of the tumors in the studied patient group exhibited massive immune cell infiltrations which might have interfered with measurement of the immunostained areas by dilution of pan-cytokeratin staining. As the mean immunostaining intensity did not exert prognostic significance, it can be concluded that the prognostic value was provided by the pan-cytokeratin amount (mean immunostaining intensity feature) rather than the amount/number of the malignant cells (total area of immunostaining feature).

Fractal and intensity immunostaining features presented similar prognostic performance. This was striking in view of the fact that intensity quantification is the standard approach to immunostaining evaluation, while fractal analysis is mainly considered as a method of choice for pan-tissue staining. We have previously conducted a fractal analysis of the pan-tissue H&E stained tumor histopathology specimens for the prognostic purpose (24, 28). Fractal analysis thereby performed far better on grayscale then on binary images (28) which directly opposed the results obtained in this study. The prognostic outperformance of binary fractal analysis achieved here was surprising because grayscale image binarization results in a massive loss of information. The observed discrepancy may be due to the difference in staining methods as our previous study examined the pan-tissue stained while our current study investigated immunostained tumor histology specimens. Therefore, the prognostically beneficial effect of binarization might derive from the clearer separation between the immunostained and unstained areas in binary images. Based on both studies, we conclude that grayscale information is of key importance for the pan-tissue stained, while binary information is the optimal source of prognostic information for the cytokeratin immunostained tumor tissue sections.

Another surprising result was that monofractal analysis prognostically outperformed its multifractal extension. This result was not expected in view of the fact that multifractal analysis was particularly designed for improved analysis of irregular natural forms with their typically uneven distribution of complexity. Our previous study has indicated a comparable prognostic value of monofractal and multifractal analyses of pan-tissue stained breast tumor tissue sections (28).

The observed association of ^*bin*^*FD* with the lower metastasis risk was in disagreement with the previous fractal analysis studies of pan-cytokeratin stained breast tumor tissue sections (14, 15). This discrepancy might be explained by differences between the used breast cancer patient groups. For instance, the current study used a group of patients without chemotherapy or other systemic treatments, while in the mentioned previous studies patient treatments were not specified. Furthermore, while this study analyzed the representative fields of view selected from whole tissue sections by the expert pathologist, in previous studies the selection was randomized.

While ^*bin*^*FD* is mainly considered as a measure of object distribution and shapes, ^*bin*^*FD*_*outline*_ is sensitive mainly to shapes as it was obtained by use of outlined images showing only borders/contours of immunostained structures. As these two fractal dimensions showed similar prognostic performance, it turns out that contour shapes evaluated by ^*bin*^*FD*_*outline*_ offered as much prognostic information as shapes together with distribution obtained by ^*bin*^*FD*. Furthermore, differential multifractal analysis calculates grayscale differences within boxes, thus providing sensitivity to immunostaining texture. Yet, due to the mentioned prognostic failure of multifractal grayscale image analysis, it is obvious that this type of texture analysis did not offer any prognostically useful clues. Taken together, it can be concluded that contour shapes of the malignant cell clusters provided the best prognostic clues.

The observed significant prognostic performance of the fractal features might thus be simply explained by their high correlation with standard intensity parameters. However, this explanation is not plausible in view of the multivariate analysis results which indicated the prognostic independence of fractal but not of intensity features.

Advantages of this study include the first exhaustive prognostic evaluation of the immunostaining intensity of breast tumor malignant cells and the immunostaining distribution, shape and texture analysis. Another major advantage was the highly homogenous patient group without systemic therapy, lymph node spread and with smaller tumor size (pT1/2). To assemble such a group, we needed to go 25 years back into archives as more recent treatment protocols prescribe systemic cytotoxic and/or hormonal treatments to most patients. Moreover, statistical reliability was enhanced by use of bootstrap as the bias-correction method. Benefits of the study design further include a 2-fold evaluation of the prognostic significance, by ROC and Cox regression analyses, followed by the multivariate analysis as an estimation of the potential clinical usefulness. The advantage of ROC analysis is in its use of continuous values which renders it independent from the bias-introducing value categorization. The limitation of the ROC analysis derives from its inability to take into account the time to metastasis. Therefore, we also employed Cox regression for prognostic evaluation. Limitations of this study included its relatively small sample size of 73 patients. However, this number by far exceeded the requirement estimated by the prospective sample size analysis and the high homogeneity of the patient group further supported the reliability of the obtained results. The limitation of the monofractal analysis to provide an insight into the regional variations of pixel distribution was overcome by the inclusion of multifractal analysis. Furthermore, the computational analysis techniques for immunostaining evaluation used in this study are fully objective. This is advantageous over the traditional subjective staining intensity scoring by a pathologist. However, the overall workflow employed in this study still included a residual subjectivity limitation at the level of selection of representative tumor histopathology areas for analysis. Another notable limitation was that pan-cytokeratin AE1/AE3 antibody cocktail immunostains both normal and malignant breast epithelial cells. This limitation was largely overcome by selection of the predominantly malignant tumor areas, based on morphological criteria. Therefore, the pan-cytokeratin staining in the current study mainly indicated the growth patterns of malignant cells.

In conclusion, we report several novel and unexpected findings highlighting the non-redundant prognostic value of shapes and distribution of malignant cell clusters in breast tumors, as assessed by fractal analysis. Prognostic advancement is clinically highly relevant based on the potential survival benefit for the reliably identified high-risk patients. Further research is underway in the direction of additional enhancement of breast tumor malignant growth analysis.

## Author contributions

NR performed fractal analysis on grayscale images and provided its interpretation. KP and XL developed a stain decomposition algorithm and were involved in interpretation of image analysis data. KK selected the representative histopathology slides and field of views, also provided the clinical interpretation of results. MR designed the study and did most of the writing and statistics. NM performed fractal analysis of binary images, provided its interpretations and a final revision of the manuscript.

### Conflict of interest statement

The authors declare that the research was conducted in the absence of any commercial or financial relationships that could be construed as a potential conflict of interest.

## Abbreviations

TNM, tumor size: lymph node and metastasis status
*FD*: fractal dimension
^*bin*^*FD*: binary fractal dimension
^*gray*^*FD*: grayscale fractal dimension
Λ: lacunarity
AUC: area under the rate of change curve
HR: hazard ratio
H&E: hematoxylin and eosin.
